# Modification of Thermal and Mechanical Properties of PEG-PPG-PEG Copolymer (F127) with MA-POSS

**DOI:** 10.3390/polym8090341

**Published:** 2016-09-15

**Authors:** Qingqing Dou, Anis Abdul Karim, Xian Jun Loh

**Affiliations:** 1 Institute of Materials Research and Engineering, A*STAR (Agency for Science, Technology and Research), Singapore 117602, Singapore; douq@imre.a-star.edu.sg (Q.D.); anisak@imre.a-star.edu.sg (A.A.K.); 2 Department of Materials Science and Engineering, National University of Singapore, Singapore 117576, Singapore; 3 Singapore Eye Research Institute, Singapore 168751, Singapore

**Keywords:** POSS, rheology, thermal responsive, mechanical property

## Abstract

Pluronic F127 exhibits thermogelling behaviour at 20–30 °C via a micelle packing mechanism. Disruption of the micelle packing increases the sol-gel temperature, but results in the decrease of modulus. Herein, we reported a method to modify F127 with polyhedral oligosilsesquioxane (POSS) to impart a higher gelling temperature without yielding the property and strength of the thermogel. The thermal degradation temperature was enhanced to 15 °C after POSS incorporation and the gelling temperature shifted 10 °C higher, without sacrificing the modulus of the gel. Rheological studies supported the claim that the gel property was reinforced after POSS incorporation. F127-POSS copolymer matrix stored more energy from POSS reinforcement, which saw larger Lissajous curve areas before the collapse of the microstructure for the same amount of stress applied. These results indicated that modification with POSS would raise the sol-gel transition temperature without sacrificing the modulus of the gel.

## 1. Introduction

Pluronic F127 is an amphiphilic block copolymer of PEG-PPG-PEG structure (PEG: poly(ethylene glycol); PPG: poly(propylene glycol)). The central PPG block becomes hydrophobic, while the PEG blocks remain hydrophilic and above a critical temperature, self-aggregating into micelles. At high enough concentration, these aggregated micelles form a gel. The use of Pluronic F127 could be vastly applied in pharmaceuticals, protein/drug delivery, and tissue engineering [1]. This triblock copolymer exhibited gelation at an elevated temperature, which increased toughness and viscosity of the polymer [2]. Loh and Wang observed a rise in viscosity from 3.6 to 5.0 Pa·s caused by increasing the Pluronic F127 mass concentration from 0%–3% in a polyvinylidene fluoride (PVDF)-doped solution [3]. These studies showed that modifications to increase the sol-gel transition temperature would simultaneously decrease the gel modulus, affecting its overall property.

Several studies reported the effect of hydrophilic/hydrophobic interactions on sol-gel transition and moduli properties. Aqueous Pluronic solutions are known to undergo sol-to-gel transition through a shift from unimer to micelle. High hydrophilic content minimized hydrophobic interactions which reduced the micelle aggregation tendency. The transformation from sol to gel would be difficult, resulting in the increase in the sol-to-gel transition temperature [4]. Kim et al. reported poly(poly((lactic acid-*co*-glycolic acid)-*block*-poly(ethylene glycol)) methacrylate (poly((PLGA-*b*-PEG)MA)) copolymer aqueous solution sol-gel transition behavior with increasing temperature [4]. As the compositions of the hydrophilic PEGMA (Poly(ethylene glycol) methacrylate) and acrylic acid (AA) increased, lesser hydrophobic interactions increased the critical micelle concentration, causing an increase in sol-gel transition temperature. Another study by Zhang et al. evaluated the sol-gel transition of mPEG-*g*-chitosan due to changes in the balance between hydrophilicity and hydrophobicity [5]. Elevated temperatures increased the thermal energy for polymer chain rearrangement and where the mPEG-*g*-chitosan sol-gel transition driven by hydrophobic interactions, the mPEG-*g*-chitosan chains formed aggregates, resulting in the formation of a gel network. A study by Kadla and Korehei explained the effect of hydrophilic and hydrophobic interactions on rheological behaviour and gel microstructure [6]. Increasing the alkyl chain length (hydrophobicity) enhanced the viscoelastic properties and resulted in a larger, more heterogeneous, network structure despite the increase in moduli.

Studies have reported on the incorporation of polyhedral oligosilsesquioxane (POSS) as physical and chemical modifiers in polymeric systems [7]. POSS is a hybrid organic-inorganic organosilicon of rigid nanocaged structure [8]. Incorporation of POSS into a linear polymer chain would affect the overall molecular nature of the host polymer. The rigidity of the POSS nanostructure would influence chain motion at the molecular level, by influencing mobility and interactions between segments. As a result, the polymer may undergo a macroscopic change in morphology or property [9]. He et al. dedicated numerous efforts on POSS-incorporated polymers. They investigated self-assembly behaviour of PEG-P(MA-POSS copolymers) synthesized via atom transfer radical polymerization [10]. They found that triblock copolymers P(MA-POSS)_4_-*b*-PEG_10K_-*b*-P(MA-POSS)_4_ were able to form a gel in water at ~8.8 wt %, while diblock copolymers cannot. Subsequently, they tailored gelation of (PEG−P(MA-POSS)) block copolymers by adding hydrophobic POSS (polyhedral oligomeric-silsesquioxane) nanoparticles [11]. Vinyl groups present in the POSS nanoparticles were able to enhance rheological properties of the hydrogels with UV treatment. He et al. also incorporated POSS in poly(ε-caprolactone) (PCLs) shape memory polymers, without affecting the shape memory, while increasing the melting temperature, as well as the melt crystallization temperature [12]. They further studied the triple-shape properties of polyurethane POSS-PCL varying the length of the PCL chain with a triple-shape functionalization process [13]. They also studied stereocomplexing drove self-assembly of diblock copolymers (PLLA-*b*-P(MA-POSS) and PDLA-*b*-P(MA-POSS)). Increasing the length of the (MA-POSS) block in the copolymer resulted in a size decrease of hybrid nanoparticles [14]. The group also demonstrated pH-responsive poly(acrylic acid)-incorporated POSS without sacrificing the intrinsic pH-dependent self-assembly behaviour [15].

This nanocaged framework makes POSS thermally and chemically robust and, thus, rendered it suitable as a viscosity modifier [9], mechanical enhancer, and flame retarder [10]. Lewicki et al., reported a series of polyurethane (PU)/POSS nanohybrid elastomers that exhibited increased thermal stability as compared to the unmodified PU matrix [16]. This, consequently, reduced the level of volatile degradation products and exhibited a 30 °C increase in onset degradation temperature, improving the processing capacity at elevated temperatures. Kaneko et al. cross-linked POSS and 3-(2-aminoethylamino)propyltrimethoxysilane with bis (3-(trimethoxysilyl)propyl)amine [17]. The copolymer exhibited high thermal stability with 5% and 10% weight losses at 351 and 368 °C, respectively. Blanco et al. copolymerized POSS with styrene and different POSS contents (3%, 5%, and 10%, *w*/*w*) [18]. They observed initial decomposition temperatures and temperatures at 5% mass loss of the copolymers, increased with increasing POSS contents. Another study demonstrated an increase in the glass transition temperature (*T*_g_) after incorporation of POSS. Wunder et al. synthesized a POSS-PEG copolymer blended LiClO_4_ and methyl cellulose to enhance the property of the solid polymer electrolytes [19]. The increase of the glass transition temperature (*T*_g_) of blends indicated the reinforcement of conductive POSS-PEG to the LiClO_4_ partitions.

Herein, we reported F127 modification by incorporation of methacryloisobutyl-POSS (MA-POSS) to enhance mechanical and thermal properties. Studies on MA-POSS-based polymers have shown effective improvement in the glass transition temperature (*T*_g_) of the polymer nanocomposite [20,21]. The degree of hydrophilicity of F127 is altered by the addition of hydrophobic MA-POSS; in small dosages MA-POSS was expected to enhance the modulus and strength of F127 while maintaining viscoelastic characteristics of F127.

Subsequently, rheological studies were performed to understand the thermal responsive behaviour of polymer at microscopic level via dynamic oscillatory shear tests. The reinforcement of rigid POSS to F127 was investigated by oscillatory amplitude sweeps in response to gelation. In addition, copolymer microstructure interactions were studied with Lissajous curves extracted from the oscillatory amplitude subjected to a sinusoidal deformation [22]. The shape evolution derived from distorted stress waveforms of Lissajous curves could be linked to microstructural change within the polymer matrix in response to stress.

## 2. Materials and Methods

Pluronic F127, Ethyl α-bromoisobutyrate (EBIB), 1,1,4,7,10,10-hexamethyltriethylenetetramine (HMTETA, 99%), copper (I) bromide (CuBr) (99%), triethylamine, 2-bromoisobutyryl bromide, tetrahydrofuran (THF) anhydrous, and ethyl ether anhydrous were obtained from Aldrich, Singapore. Methacrylisobutyl POSS (MA-POSS) was purchased from Hybrid Plastics, Hattiesburg, US (product #: MA0702). All chemicals were used as received without further purification. Purified nitrogen was used in all of the reactions.

### 2.1. Synthesis of F127-POSS Copolymers by Atom Transfer Radical Polymerization (ATRP)

F127 initiator for ATRP was synthesized according to previous report [23]. F127 (10 g, 0.79 mmol) was dissolved in 50 mL of anhydrous THF in a 250 mL round-bottom flask and kept in an ice/water bath, and 0.3314 mL of triethylamine was added. Subsequently, 0.59 mL of 2-bromoisobutyl bromide was added dropwise into the flask through an equalizing funnel. The bromination reaction mixture was stirred at room temperature for 24 h. The resulting Br-F127-Br macroinitiator was precipitated in excess diethylether/methanol (80:20 *v*/*v*). The crude product was purified by dissolving in tetrahydrofuran (THF) and any residual reactants were removed by precipitation twice with hexane and dried in vacuum for subsequent ATRP. F127-poly(methacrylisobutyl POSS) (F127-*p*-MA-POSS) copolymers were synthesized using two different molar feed ratios of F127:MA-POSS, 1:4 and 1:8, respectively. As mentioned, hydrophilic property of F127 would be altered by the addition of hydrophobic MA-POSS. Therefore, to ensure minimal solubility of polymer in water the maximum ratio of POSS to be added to F127 was investigated to be 1:8 (F127:MA-POSS). The feed amount used for each synthesis is listed in Table 1. Br-F127-Br, MA-POSS, and EBIB were mixed and dissolved in 15 mL 2-propanol at room temperature. The mixture was then degassed with nitrogen for 10 min to remove the oxygen scavenger. 28 mg of CuBr and 0.107 mL of HMTETA were then added to the reaction mixture under nitrogen atmosphere. The reaction mixture was further purged with nitrogen for 10 min and sealed. The polymerization was allowed to proceed under continuous stirring at 40 °C for 24 h. The reaction was quenched by diluting with THF and exposing the reaction mixture to air. The catalyst complex was removed by passing the polymer solution through a short aluminum oxide column. THF was then removed from the solution by rotary evaporation under reduced pressure. Any unreacted monomer was removed by precipitation with hexane. The obtained precipitate was then dissolved in THF, precipitated once again in hexane, and dried under vacuum overnight.

### 2.2. Molecular Characterisation

Characterizations of the polymers were carried out as previously reported [24,25]. ^1^H NMR (400 MHz) was used to determine the structures of polymers synthesized. The spectra were recorded with a Bruker AV-400 NMR spectrometer (Bruker, Billerica, MA, USA) in chloroform-d (CHCl_3_) at room temperature. A 30° pulse width, 5208 Hz spectral width, and 32,000 data points were used for the measurement. The acquisition time was set to 3.2 s with a pulse repetition time of 2.0 s. Chemical shift was normalized to the solvent peaks (δ = 7.26 ppm for CHCl_3_). Molecular weight was measured with gel permeation chromatography (GPC, Waters 2690, Milford, MA, USA.) with a Shimadzu SCL-10A and LC-8A system, equipped with a Shimadzu RID-10A refractive index detector (Shimadzu, Chiyoda-ku, Tokyo, Japan) and two 5 μm Phenogel 50 and 1000 Å columns (size: 300 × 4.6 mm) in series. In both measurements, THF was used as eluent at 40 °C, and flow rate of 0.30 mL·min^−1^. The calibration curve was performed with monodisperse polystyrene standards.

### 2.3. Thermal Degradation Characterisation

Thermogravimetric analysis (TGA) was performed on a TA Instruments SDT 2960 (TA Instruments, New Castle, DE, USA). Samples were heated from 30–800 °C with heating rate of 20 °C·min^−1^ under dynamic nitrogen flow at 70 mL·min^−1^.

### 2.4. Rheological Measurements

Rheological tests were performed with a Discovery Hybrid Rheometer (DHR-3, TA Instruments-Waters L.L.C., New Castle, DE, USA) fitted with a 20 mm parallel plate geometry. Temperature was regulated by a Peltier plate system used to report temperature-responsive behaviour of the polymer. Temperature sweep tests were performed with an oscillation temperature ramp of 25–45 °C at a ramp rate of 1 °C·s^−1^, at constant strain of 1.25%, and angular frequency of 10 rad·s^−1^. Dynamic oscillatory amplitude tests were performed by varying the strain from 0.5%–150% at 25 and 30 °C, respectively, at constant frequency of 1 Hz. Lissajous curves were recorded with a sampling time of 3 s. A solvent-trap cover (TA Instruments) was applied during each test to prevent water evaporation.

## 3. Results

### 3.1. Synthesis of Polymers

**Figure 1:**
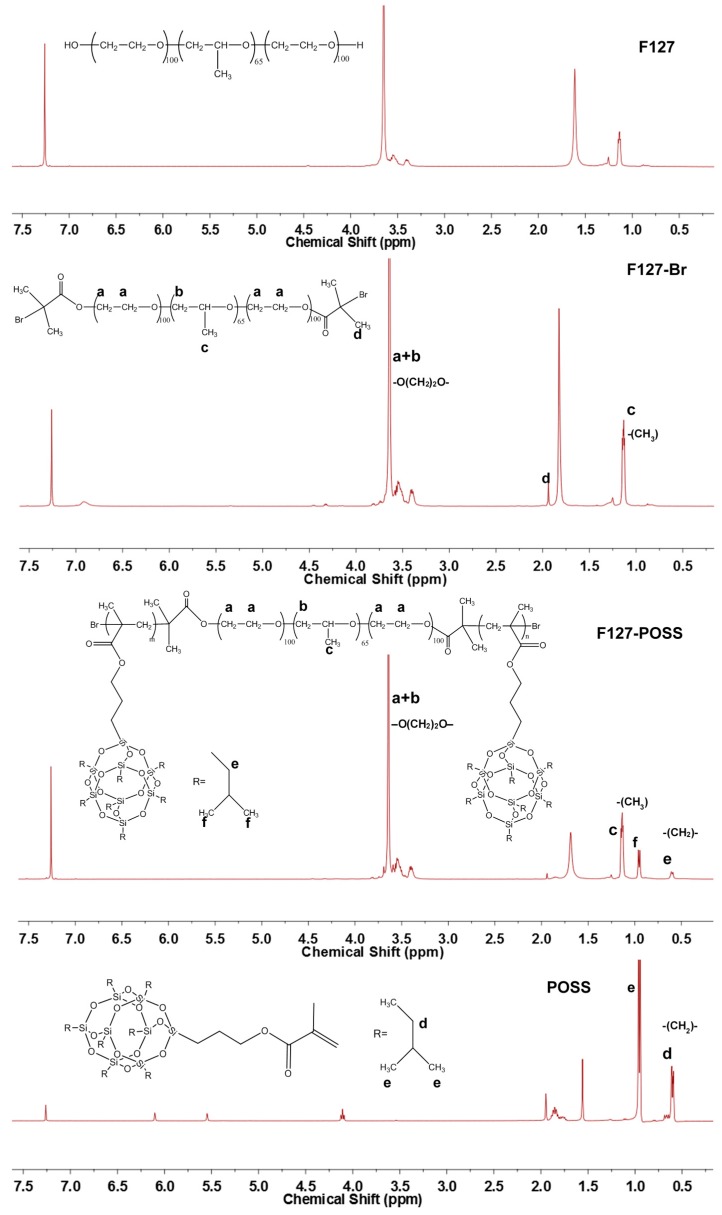
^1^H NMR spectra of F127, F127-Br, F127-POSS, and methacrylisobutyl POSS.

**Scheme 1:**
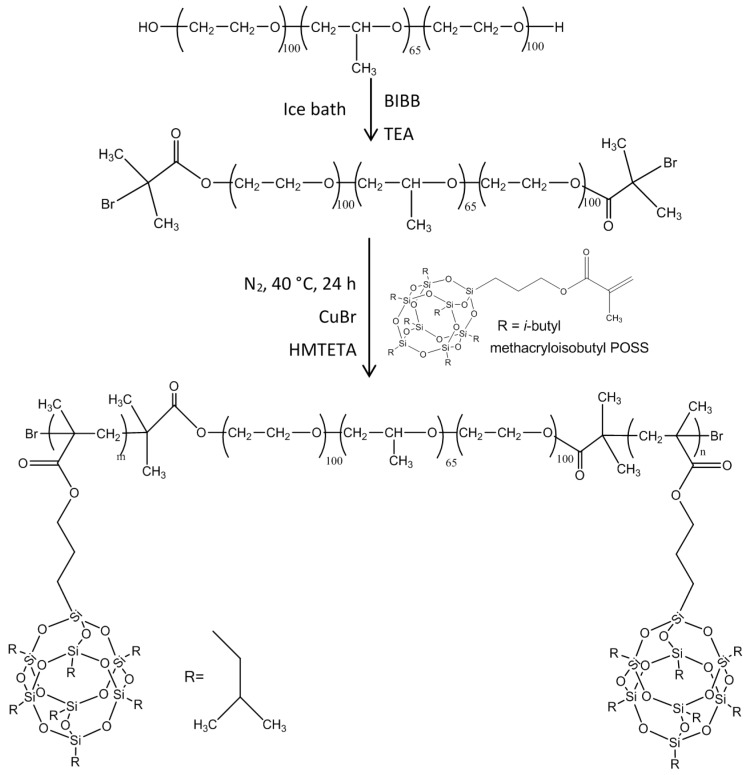
Synthesis procedure for F127-POSS.

The polymer was synthesized as shown in Scheme 1. F127 was modified to introduce –Br active ends by bromination with BIBB in the presence of triethylamine (TEA) in an ice bath. A chemical shift at 1.94 ppm in the ^1^H NMR spectra (in CDCl_3_, Figure 1) proved that 97.1% of F127 was successfully converted to the Br-F127-Br initiator. Subsequently, methacrylisobutyl polyhedral oligomeric silsesquioxane (MA-POSS) was polymerized at the ends of the F127 initiator through atom transfer radical polymerization (ATRP) in two ratios of F127:MA-POSS, 1:4 or 1:8. The ratio of hydrophobic POSS added had altered the hydrophilic property of F127, with a ratio of 1:8 (F127:MA-POSS) showing partial solubility in water, impeding usage in water-based applications. Therefore, subsequent studies focused on F127-POSS with a ratio of 1:4, which could be completely dissolved in water. ^1^H NMR (in CDCl_3_, Figure 1) indicated successful polymerization between MA-POSS and F127. δ = 3.64 ppm depicted a proton signal of (–O(CH_2_)_2_–O–) together with (–O–CH_2_–) in the F127 backbone. –CH_3_ groups of MA-POSS could be found around 1.13 ppm. A slight variation in signals could be related to the amount of MA-POSS present in the sample. A slight downward shift in signal could be observed as the degree of MA-POSS increased in the sample. Resonance peaks observed at δ ~0.94–0.96 and 0.609 ppm could be assigned to –CH_3_ and –CH_2_– groups of isobutyl of MA-POSS, respectively [26,27]. With the integration ratio of resonance peaks of methylene groups in the F127 backbone (3.64 ppm) and methylene groups in MA-POSS (0.609 ppm), the molar ratio of MA-POSS to F127 was found to be 2:1. The integration ratio of methyl groups in F127 backbone to methyl groups in MA-POSS also depicted two MA-POSS was attached on one F127 polymer chains.

### 3.2. Thermal Degradation Analysis

**Figure 2:**
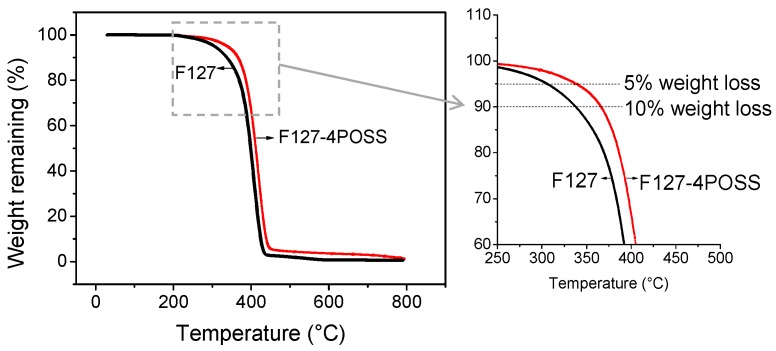
Thermal decomposition analysis of F127 and F127-POSS by TGA.

TGA was used to compare thermal degradations of F127 precursor and F127-POSS over a temperature range of 30–800 °C, at a rate of 20 °C·min^−1^ in nitrogen atmosphere (Figure 2). The onset thermal degradation (defined as 5 wt % loss) of F127-POSS were observed at 339.6 °C, which was 34.0 °C higher than pure F127 (305.6 °C, Table 2). Lewicki et al. reported PHIPOSS/polyurethane nanohybrid elastomers exhibited an increased 10 °C onset degradation temperature after 10% loading of POSS [16]. Our F127-POSS results showed much higher increment of degradation temperature compared to the PHIPOSS/polyurethane. The temperature difference became apparent with higher weight loss at elevated temperatures. It showed 28.3 °C higher over F127 with 10% weight loss, 366.0 and 337.7 °C, respectively. Moreover, the temperature of the maximum rate of weight loss can be found from the first derivative of the TGA curve (Figure S1), which was 417.6 °C for F127-POSS and 403.9 °C for F127. Up to 800 °C, F127 showed 0.6 wt % remaining, while F127-POSS showed 1.2 wt % leftover, white charred residues in the TGA crucibles. Analysed together with the NMR results, the leftover residual was confirmed to be un-degradable Si in MA-POSS. Therefore, incorporation of POSS increased the degradation temperature (i.e., delaying degradation of components) due to the rigidity of the nanosize-block POSS which restricted the polymer chain motion.

### 3.3. Thermal Gelling Behaviour

The thermogelling property of F127 aqueous solution has attracted significant interest involving drug release applications [28,29,30,31,32,33]. This intrinsic property was induced by the PPG segment in F127 [33,34,35,36]. A hydration layer surrounding F127 via hydrogen bonds sustained solvation of the polymer; however, at elevated temperature, water-polymer interactions weakened resulting in the association of the hydrophobic PPG components, leading to gelation [37]. The gelation temperature could be elevated in the presence of the rigid POSS structure. This rigid nano-block would hinder the association of the PPG hydrophobic segments which induced gelation, increasing the gelation temperature of F127-POSS as compared to pure F127.

**Figure 3:**
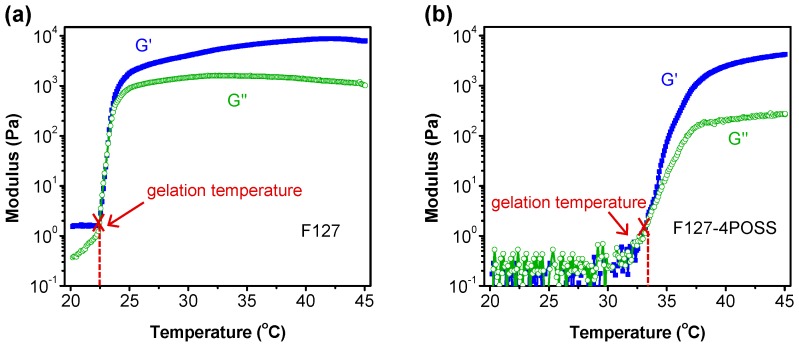
Oscillatory temperature sweep showed storage (*G*’) and loss modulus (*G*’’), and gelation temperature of 15 wt % (**a**) F127 and (**b**) F127-POSS, between 20–45 °C, at a ramp rate of 1 °C·min^−1^, under constant strain of 1.25%, and angular frequency of 10 rad·s^−1^.

Thermogelling behaviour was investigated by conducting rheological studies on 15 wt % aqueous solutions of F127 precursor and F127-POSS. Oscillation temperature sweeps were carried out to determine the gelling temperature of the polymers; conditions set between 20–45 °C, at a ramp rate of 1 °C·min^−1^, under constant strain of 1.25%, and angular frequency of 10 rad·s^−1^. A solvent trap was applied to minimize water evaporation. It was observed that POSS incorporation on F127-POSS yield comparable gel strength (storage modulus, *G*’) to that of F127 (Figure 3). However, the incorporation of POSS shifted gelation temperature higher from 23.5 to 33.5 °C. This extended the temperature range in which it remained liquid before gelation sets in. Trace water loss at the fringe of the sample in between the two parallel plates at higher temperature was non-avoidable, despite the protection of the cover. Vapour circulated in the chamber maintained the test environment equilibrium. However, it did not affect the comparison of the two samples due to the identical test protocol used for the F127-POSS and F127. The slightly higher concentration resulted from water loss might induce a higher modulus. The modulus finally stabilized at a plateau, indicating the water evaporation did not affect the test results by much.

Oscillatory amplitude sweeps were performed on 15 wt % aqueous solutions of F127 and F127-POSS at two different temperatures (25 and 30 °C) to study the effect of POSS incorporation under linear viscoelastic region (LVE) [38,39,40]. Further in-depth analysis on oscillatory shear tests would provide more information on the microstructure when the material was subjected to a sinusoidal deformation measured by the resulting mechanical response as a function of time [22]. As the applied amplitude (of strain or stress) increased from small to large at a fixed frequency during oscillatory shear tests, two regions were defined: (1) small amplitude oscillatory shear test (SAOS); and (2) large amplitude oscillatory shear test (LAOS) (Figure S2). In SAOS, the *G*’ and *G*” moduli were independent of the applied strain amplitude at a fixed frequency and the resulting stress was a sinusoidal wave, defined as the linear viscoelastic region (LVE). In LAOS, the *G*’ and *G*” moduli became a function of the strain amplitude at a fixed frequency and the resulting stress waveforms were distorted from sinusoidal waves, defined as the non-LVE region [41,42]. This region is of great interest, as distortion from sinusoidal waveforms resulted in nonlinear moduli data to understand the mechanism related to microstructural change of the material.

**Figure 4:**
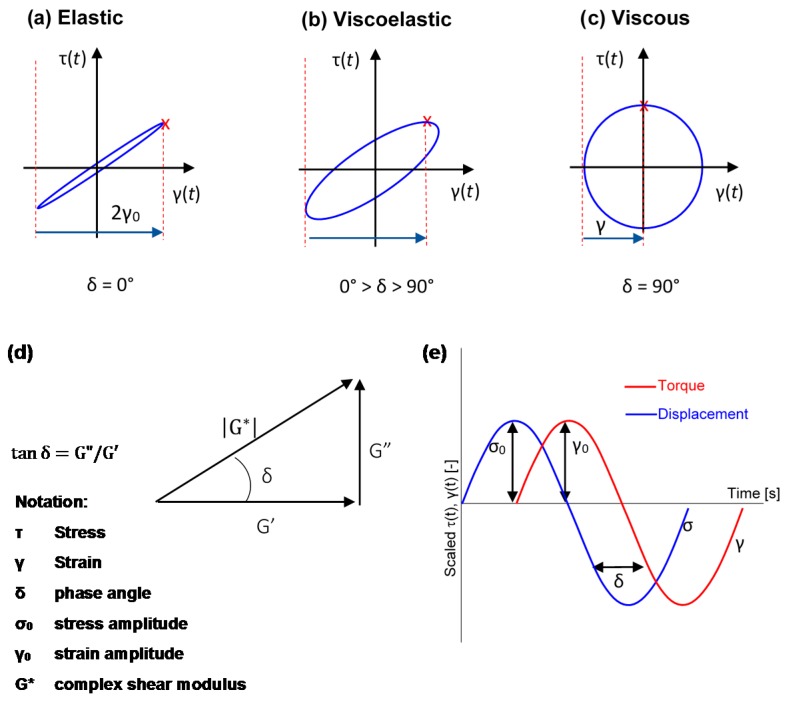
Elastic Lissajous–Bowditch representations (stress plotted against strain) of three ideal material responses, (**a**) the point of greatest stress occurs at 2γ_0_ after the previous strain reversal; (**b**) linear viscoelastic response (center) has the stress maximum somewhere intermediate to these limits; (**c**) the stress maximum achieved at the point where the material has acquired γ_0_ strain since the previous reversal in an ideal viscous material [44]. Reproduced from McKinley et al. [45]; (**d**) vectorial representation of moduli; and (**e**) sinusoidal wave forms for stress and strain functions. Reproduced from Edward et al. [46].

The oscillatory stress sweeps data could extract additional information represented by Lissajous curves to further analyse the microstructure of polymer. Lissajous curves (as shown in Figure 4) are closed-loop graphical representations of stress (*y*-axis) vs. strain/strain rate (*x*-axis), used to predict the generic behavioural mechanism of deformation [43]. Lissajous curves showed three distinct shapes corresponding to phase change. This fingerprint graphical plot is formed when two harmonic vibrations of stress vs. shear strain or shear-rate along perpendicular lines are superimposed. When strain amplitude is large, the stress becomes no longer sinusoidal and has higher harmonic contributions, where the strain magnitude (γ) and the phase angle (δ) depend on strain amplitude and imposed frequency (ω) [42]. A transition between the linear and nonlinear regimes was observed with the change in shape of Lissajous curves from elliptical to rectangular in the LAOS test.

**Figure 5:**
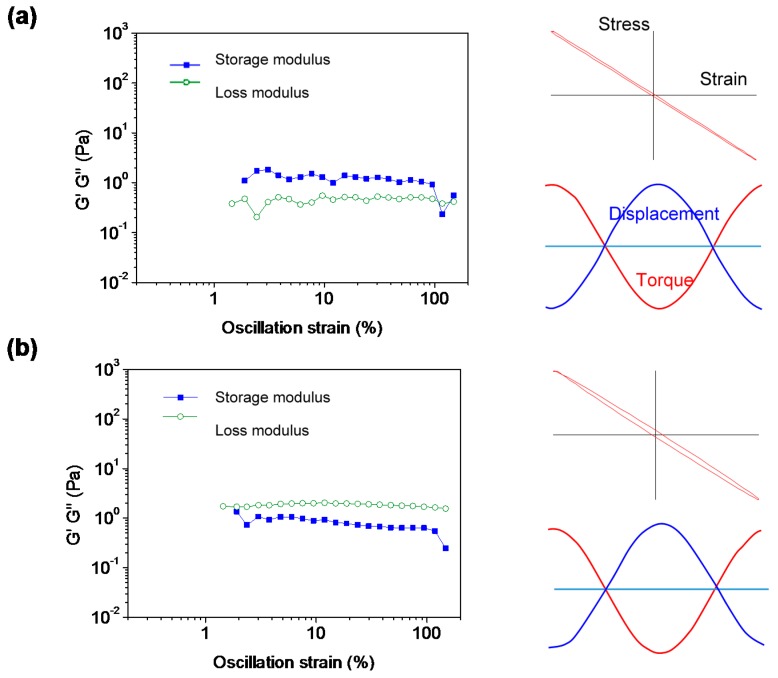
Oscillatory amplitude sweep of 15 wt % polymers at 25 °C; (**a**) F127; and (**b**) F127-POSS, and corresponding sinusoidal waveforms. The tests were performed at a strain percent range of 0.5%–150%, with a fixed frequency at 1 Hz.

Oscillatory amplitude sweeps performed at 25 °C for both F127 and F127-POSS showed steady linear viscoelastic properties within the applied strain range of 0.5%–150% (Figure 5). This implied that the incorporation of POSS did not alter the flow/deformation behaviour of the material and its internal structure at room temperature. It is worthy to note that the low moduli values around 1 Pa were indicative of a liquid state even though Figure 5a suggested F127 showed slightly larger *G*’ than *G*”, but the difference in *G*’ and *G*” is minute (this similar trend could be explained for Figure 3a). Both F127-POSS and F127 exhibited steady, but very low moduli (around 1 Pa), at 25 °C. This corresponded to the stress-strain Lissajous curves depicted by narrow elliptical form as shown in Figure S3 over the entire oscillatory amplitude sweep. At 25 °C, displacement waveform displayed 180° out of phase with torque throughout the measurement, indicating the liquid form of sample [47].

**Figure 6:**
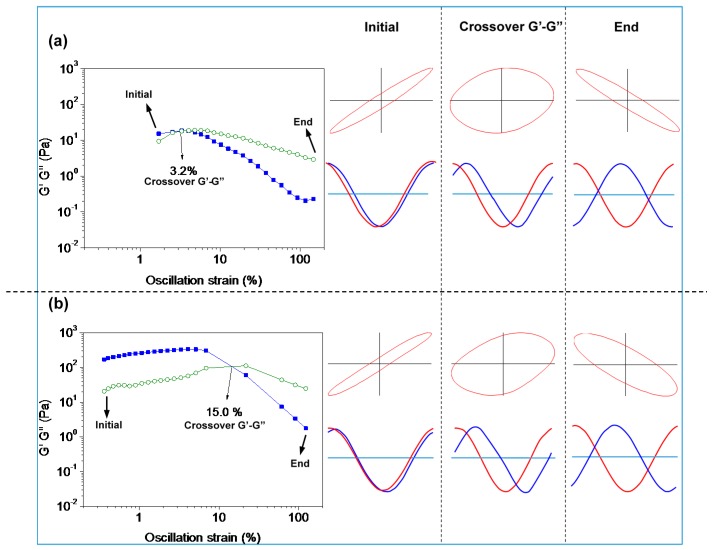
Oscillatory amplitude sweep of 15 wt % polymers at 30 °C; (**a**) F127, and (**b**) F127-POSS, and corresponding sinusoidal waveforms. The tests were performed at a strain prcent range of 0.5%–150%, with a fixed frequency at 1 Hz.

At 30 °C, both F127 and F127-POSS undergo gel transition within a 10–100 Pa moduli range (Figure 6). With increasing strain applied to the system, it would reach a point where the gels would not be able to withstand the applied force and started to collapse, where *G*” would dominate *G*’. F127-POSS showed higher tolerance to deformation than F127, yielded at 15.0% and 3.2% strain, respectively. Incorporation of POSS has enhanced the breaking strain; increasing the hydrophobicity of the polymer matrix enhanced the viscoelastic properties. The rigid POSS structure increased the overall moduli, with moduli of F127-POSS one order higher than F127. This substantiated the assumption that addition of POSS reinforces the strength of F127.

Three points were selected for Lissajous curves as shown in Figure 6; initial point, phase transition crossover point (*G*’-*G*”), and the end point. The different Lissajous shapes indicated a microstructural change of the samples during the oscillatory amplitude sweep. The area enclosed by the Lissajous curve corresponded to the energy dissipated per unit volume sample in a single LAOS cycle [48]. At low strain, a soft gel was formed as the strain decreased immediately with increasing stress, indicating a viscoelastic property (narrow elliptical). The Lissajous shape transformed to rectangular with a larger area at the phase transition point, inferring more energy being dissipated in the sample. Eventually, the sample could not hold more energy, whereby the internal forces induced by higher strain would break the bonds, resulting in collapse of the gel microstructure, as reflected by the decreased ellipse area. Comparing F127-POSS and F127 at 30 °C, the Lissajous curve area of F127-POSS was much larger compared to F127 (most prominent in the endpoint Lissajous curves), indicating F127-POSS dissipated more energy than F127. The strength of POSS-incorporated gel was enhanced; F127-POSS polymer matrix stored more energy as compared to F127 before the collapse of the microstructure for the same amount of stress applied. This further validated the theory that POSS reinforced the overall gel property. The rheological analysis was listed in Table 3 for comparison and easier understanding.

## 4. Conclusions

In this work, MA-POSS was used to modify the thermal and mechanical properties of F127 with the addition of trace amounts of POSS. MA-POSS was successfully incorporated in F127 via atomic transfer radical polymerization (ATRP). Thermogravimetric analysis depicted the onset of thermal decomposition temperature of F127-POSS was enhanced 15 °C compared to F127. The weight loss of 5% and 10% temperature was increased 34.0 and 28.3 °C respectively after POSS incorporation. Subsequently, rheological studies were performed to investigate the gel property affected by POSS incorporation. Thermal gelation of F127-POSS composite solution shifted 10 °C higher, from 23.5 to 33.5 °C, than F127 precursor, while the modulus of the gel was well maintained. A stress-strain Lissajous curve extracted from oscillatory amplitude test was used to investigate the change in the gel microstructure subjected to oscillatory strain. The area enclosed by the Lissajous curve changing with strain revealed the energy dissipated per unit volume sample, to further reflect the strength change of the gels and the molecular interaction in the gel with strain. In summary, trace amounts of MA-POSS incorporation in F127 reserved the intrinsic thermal responsive gelation property of F127 and was able to shift the gelation temperature 10 °C higher, while the strength of the hydrogel was not sacrificed. Moreover, the thermal degradation temperature was successfully extended up to 34.0 °C. It was also reported that POSS incorporation induced a polymer antibacterial property [49], which will benefit food and cosmetics applications. This profile should be investigated in subsequent studies.

## Figures and Tables

**Table 1:** Feed amount for synthesis of F127-POSS.

Sample	Initiator Br-F127-Br	Monomer MA-POSS	Ligand HMTETA	Catalyst CuBr
F127-4POSS	1.25 g	0.3909 g	0.107 mL	28 mg
F127-8POSS	1.25 g	0.7819 g	0.107 mL	28 mg

**Table 2:** Thermal degradation analysis of F127-POSS and F127.

Samples	Onset, 5 wt % loss (°C)	10 wt % loss (°C)	Max degradation rate (°C)	Weight remaining
F127-POSS	339.6	366.0	417.6	1.2 wt %
F127	305.6	337.7	403.9	0.6 wt %

**Table 3:** The summary of rheological analysis.

Tests performed	Polymer composition		Sinusoidal waveform/Lissajous curves	Inference
	F127 (Control)	F127 (Control)		
**Oscillation temperature sweep**	Gelation Moduli range: 10^3^ Pa T_Gelation_: 23.5 °C	Gelation Moduli range: 10^3^ Pa T_Gelation_: 33.5 °C		In the presence of POSS, the gelation temperature was elevated, without compromising the gel strength (modulus range)
**Oscillation amplitude sweep**	At 25 °C Moduli range: 1 Pa; No *G*’-*G*” crossover point	At 25 °C Moduli range: 1 Pa; No *G*’-*G*” crossover point	Narrow elliptical form, displacement waveform 180° out of phase with torque throughout entire oscillatory amplitude sweep, indicating liquid form	Modulus increased when temperature increased from 25 to 30 °C—indicative of the temperature-responsive property not altered for POSS-reinforced polymer. The *G*’-*G*” crossover point increased from 3.2% (F127) to 15.0% (F127-POSS), suggested F127-POSS matrix dissipated more energy as compared to F127 before the collapse of the microstructure for the same amount of stress applied
	At 30 °C Moduli range: 10^−1^–10^1^ Pa Crossover *G*’-*G*”: 3.2% strain	At 30 °C Moduli range: 10^0^–10^2^ Pa Crossover *G*’-*G*”: 15% strain	F127-POSS larger curve area compared to F127 (most prominent in endpoint Lissajous curves), indicating F127-POSS dissipated more energy than F127	

## Supplementary Materials

The following are available online at www.mdpi.com/2073-4360/8/9/341/s1. Figure S1: First derivative of TGA curves. Figure S2: Schematic illustration of the oscillation strain sweep test at a fixed frequency of 10 Hz. This sweep test was used for determining the linear and non-LVE region. In the linear region, the storage (G′) and loss (G″) moduli were independent of the applied strain amplitude at a fixed frequency and the resulting stress was a sinusoidal wave. However, in the nonlinear region, the storage and loss moduli became a function of the strain amplitude at the fixed frequency and the resulting stress waveforms were distorted from sinusoidal waves. In the linear region, the oscillatory shear test was called SAOS (small amplitude oscillatory shear), and the application of LAOS (large amplitude oscillatory shear) resulted in a nonlinear material response. Figure S3: Lissajous curve of F127-POSS and F127 extracted from oscillation amplitude sweep at 25 °C.
