# Supplementary material for: Modification of Thermal and Mechanical Properties of PEG-PPG-PEG Copolymer (F127) with MA-POSS
Source: Polymers (Basel). 2016 Sep 15;8(9):341. doi: 10.3390/polym8090341 (PMC6432308; doi:10.3390/polym8090341)
Supplement: Supplementary file 1 [file polymers-08-00341-s001.pdf]

Supplementary Materials: Modification of Thermal and Mechanical Properties of PEG-PPG-PEG Copolymer (F127) with MA-POSS

Qingqing Dou, Anis Abdul Karim and Xian Jun Loh

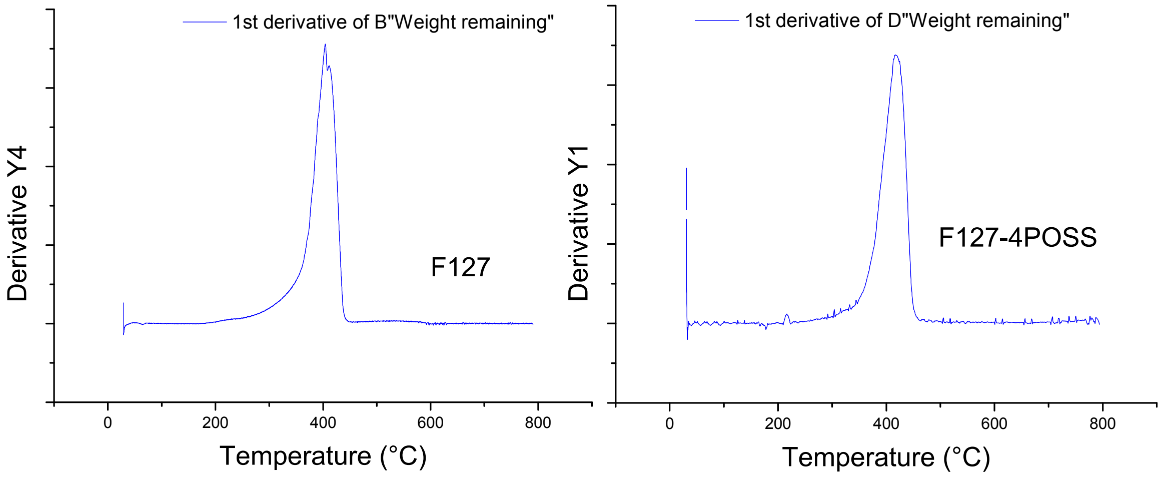

Figure S1. First derivative of TGA curves.

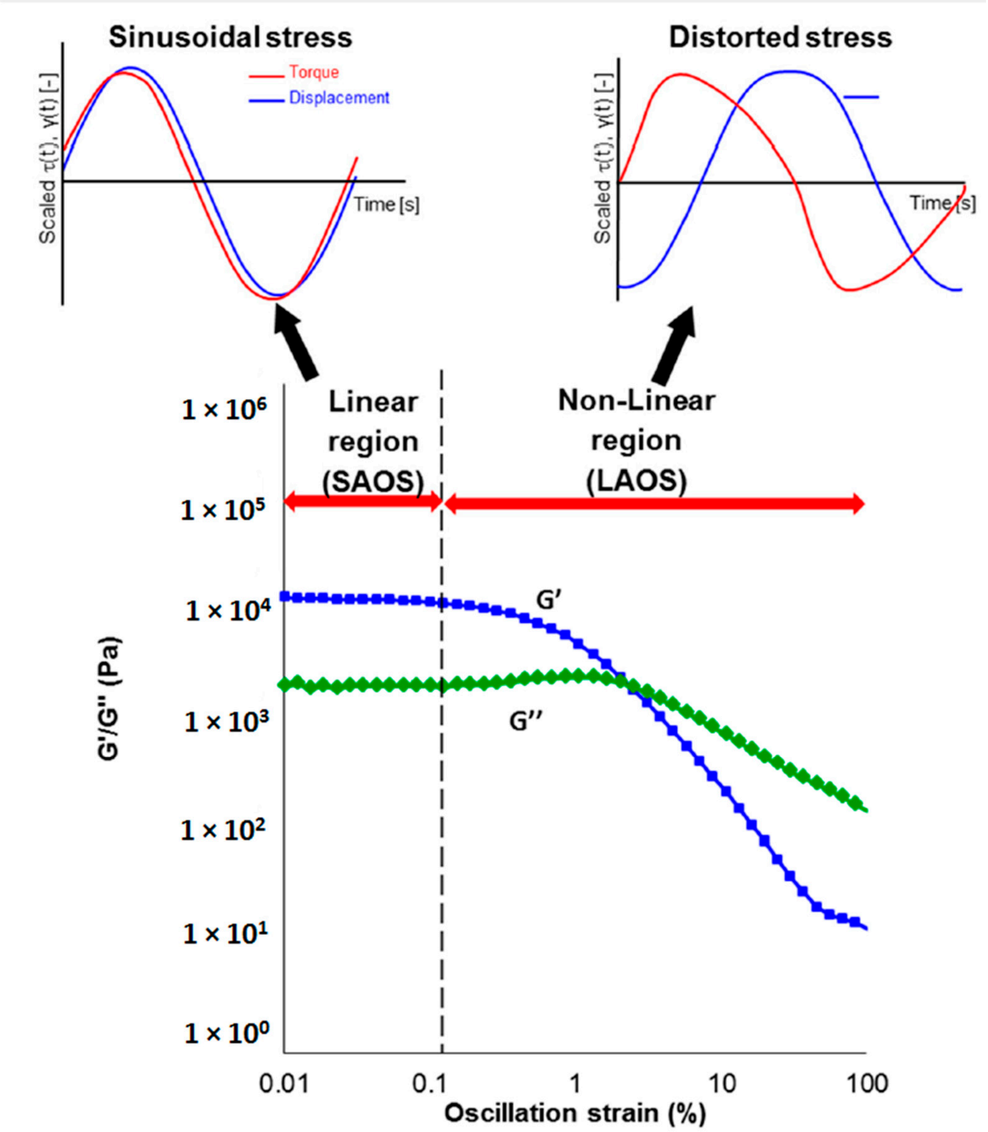

Figure S2. Schematic illustration of the oscillation strain sweep test at a fixed frequency of 10 Hz. This sweep test was used for determining the linear and non-LVE region. In the linear region, the storage (G') and loss (G'') moduli were independent of the applied strain amplitude at a fixed frequency and the resulting stress was a sinusoidal wave. However, in the nonlinear region, the storage and loss moduli became a function of the strain amplitude at the fixed frequency and the resulting stress waveforms were distorted from sinusoidal waves. In the linear region, the oscillatory shear test was called SAOS (small amplitude oscillatory shear), and the application of LAOS (large amplitude oscillatory shear) resulted in a nonlinear material response.

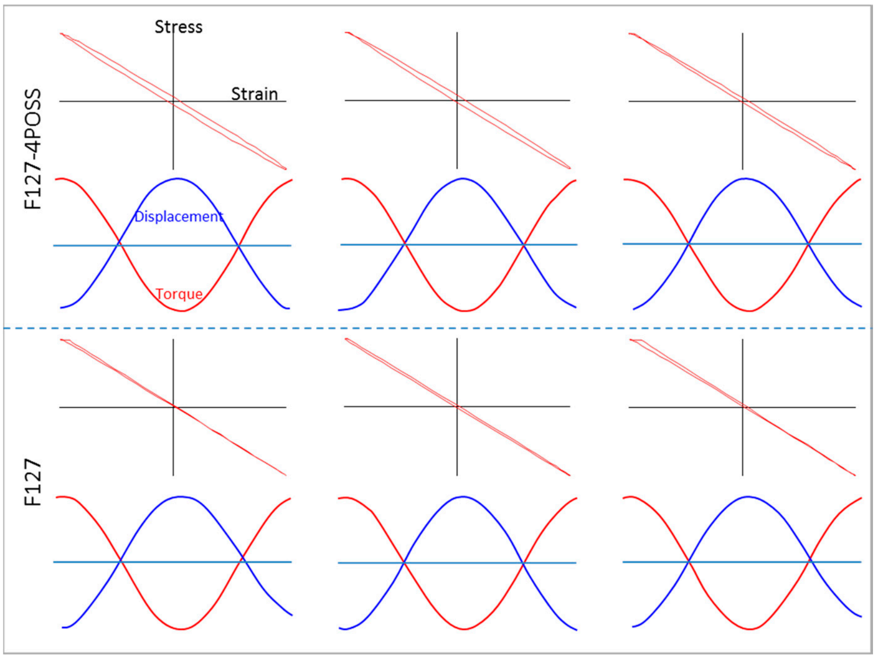

Figure S3. Lissajous curve of F127-POSS and F127 extracted from oscillation amplitude sweep at 25°C.

© 2016 by the authors; licensee MDPI, Basel, Switzerland. This article is an open access article distributed under the terms and conditions of the Creative Commons Attribution (CC-BY) license (http://creativecommons.org/licenses/by/4.0/).
